# Clinical Characteristics, Treatment, and Prognostic Factors of Patients With Primary Extramammary Paget's Disease (EMPD): A Retrospective Analysis of 44 Patients From a Single Center and an Analysis of Data From the Surveillance, Epidemiology, and End Results (SEER) Database

**DOI:** 10.3389/fonc.2020.01114

**Published:** 2020-08-25

**Authors:** Shanshan Weng, Ning Zhu, Dan Li, Yurong Chen, Yinuo Tan, Jiaqi Chen, Ying Yuan

**Affiliations:** ^1^Department of Medical Oncology, Key Laboratory of Cancer Prevention and Intervention, Ministry of Education, The Second Affiliated Hospital, Zhejiang University School of Medicine, Hangzhou, China; ^2^Department of Medical Oncology, Zhuji People's Hospital of Zhejiang Province, Shaoxing, China; ^3^Cancer Institute, Key Laboratory of Cancer Prevention and Intervention, Ministry of Education, The Second Affiliated Hospital, Zhejiang University School of Medicine, Hangzhou, China

**Keywords:** extramammary Paget's disease (EMPD), surveillance, epidemiology, end results (SEER), recurrence, prognosis, survival

## Abstract

**Objective:** Primary extramammary Paget's disease (EMPD) is a rare cutaneous malignancy. The aim of this article is to analyze clinical characteristics, evidence of the prognosis, and share treatment experience of primary EMPD.

**Methods:** We extracted 771 patients' data from the Surveillance, Epidemiology, and End Results (SEER) program between 1973 and 2013 to investigate the characteristics and prognosis of patients with EMPD. In addition, 44 patients with primary EMPD in our hospital were retrospectively reviewed for 10 years.

**Results:** Compared with patients younger than 65 years, patients diagnosed at 65–74 years [hazard ratio (HR), 2.453] and 75 years or older (HR, 5.750) had shorter survival. Patients with a primary site in the truncal skin (HR, 0.367) or scrotum (HR, 0.246) had better survival compared to those with a primary site in the perianal area. Compared with localized EMPD, EMPD with distant (HR, 18.821) and regional (HR, 2.180) metastases was associated with a worse prognosis. Patients who received radiotherapy had decreased survival, with an HR of 2.039. Patients with a higher N stage, M stage, and American Joint Committee on Cancer (AJCC) stage had a decreased prognosis (*p* < 0.001).

**Conclusions:** Older age at diagnosis, primary site in the perianal area, distant metastasis, radiotherapy, and higher N stage, M stage, and AJCC stage may result in decreased survival.

## Introduction

Primary extramammary Paget's disease (EMPD) is an uncommon malignant tumor arising in areas rich in apocrine glands such as the vulva, scrotum, perianal region, and penile skin, and common clinical symptoms are pruritus, rash, erythema, erosion, pain, and exudation (1–4). In 1874, Sir James Paget was the first to describe the pathologic features of the areolar tissue of a patient with breast cancer (5). Thus, Paget's disease was named after him. Then, in 1889, Dr. Crocker reported the first case of EMPD (6). Due to the rarity of the disease, our current knowledge about EMPD is based on a limited number of case series, and some controversies exist, such as pathogenic differences between primary and secondary EMPD, the optimal treatment for patients with recurrent EMPD, the factors affecting prognosis, and the clinical significance of human epidermal growth factor receptor 2 (HER2) overexpression. Our current study aims to analyze clinical characteristics and evidence of the prognosis based on data from the Surveillance, Epidemiology, and End Results (SEER) database and share treatment experience of primary EMPD in our center.

## Materials and Methods

**Figure 1:**
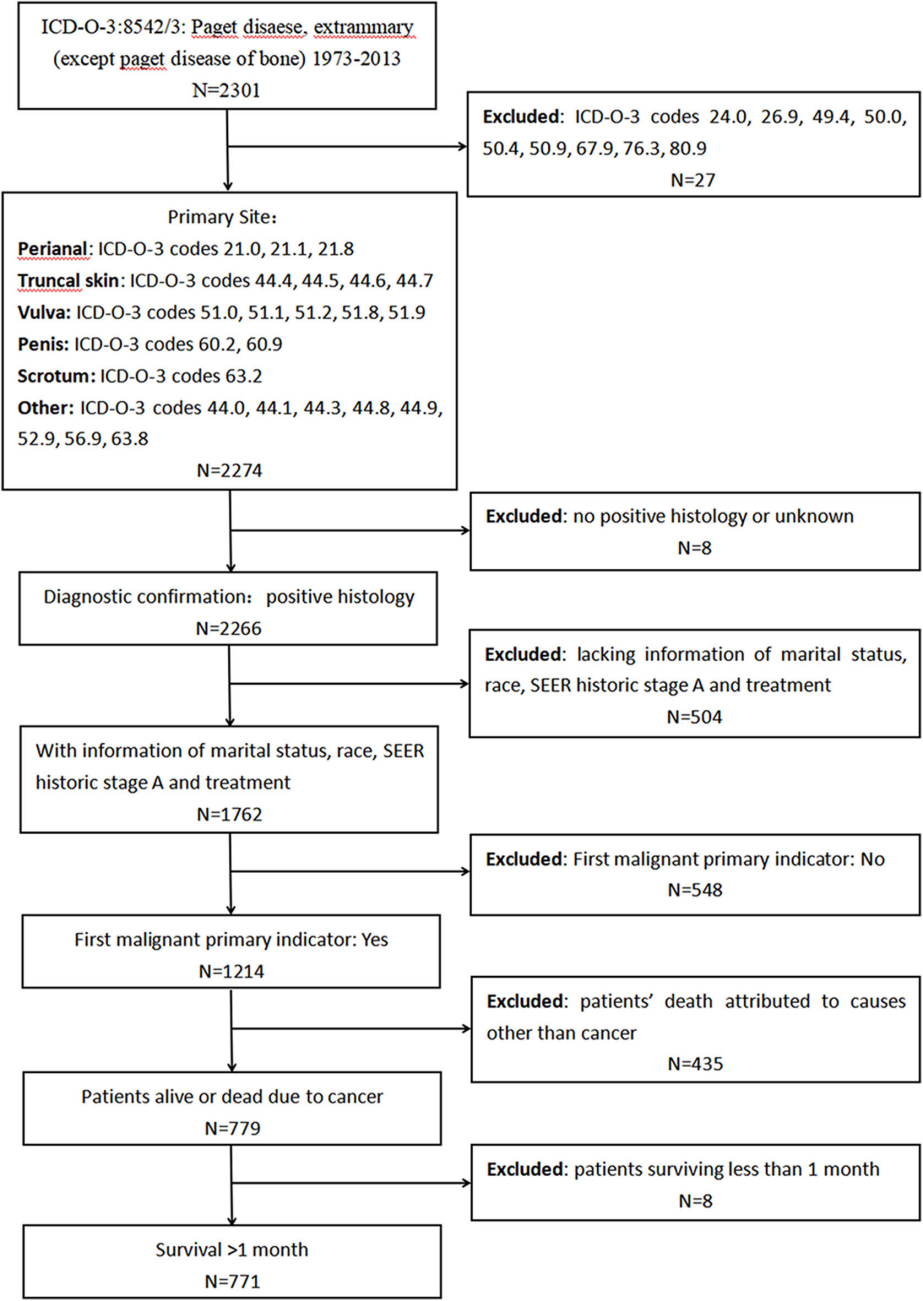
Screening process for the data from the Surveillance, Epidemiology, and End Results (SEER) program.

First, we extracted data from the SEER program to investigate the characteristics and prognosis of patients with EMPD. Data about marital status, age at diagnosis, sex, race, primary site, SEER historic stage adjustments (A), surgical treatment, and radiotherapy were analyzed. We included patients diagnosed with primary EMPD [International Classification of Diseases for Oncology, Third Edition (ICD-O-3): 8542/3] with the primary site in the perianal area, truncal skin, vulva, penis, scrotum, or other between 1973 and 2013. The screening process is shown in Figure 1. The exclusion criteria were as follows: (1): no positive histology or unknown diagnostic confirmation; (2) patients lacking documentation of marital status, race, SEER historic stage A, and treatment; (3) First malignant primary indicator: No; (4) patients whose deaths were attributed to causes other than cancer; and (5) patients who survived <1 month (in order to exclude the patients lost to follow-up).

As a supplement, demographic and clinical characteristics data of 44 patients histopathologically diagnosed with primary EMPD were retrospectively reviewed from the Second Affiliated Hospital of Zhejiang University School of Medicine records over a 10-year period from January 2008 to December 2017.

SEER^*^STAT version 8.3.5 (Surveillance Research Program, NCI, Bethesda, MD, USA) was used to extract case-level data from the SEER public-use database. All analyses were conducted with the Statistical Package for the Social Sciences (SPSS, V21.0). Kaplan–Meier methods with the log-rank test and Cox proportional hazards modeling were employed for the survival analyses. χ^2^ and Fisher's exact tests were used for the analysis of categorical variables. *P* < 0.05 was considered statistically significant. Stata 13.1 was used to calculate the hazard ratio (HR) for the stratified analysis, and GraphPad Prism 7 was used to make a forest plot.

The study was approved by the Ethics Committee of the Second Affiliated Hospital, Zhejiang University School of Medicine.

## Results

### Data From the Surveillance Epidemiology and End Results Program

#### Incidence and Demographic Characteristics

In total, from 1973 to 2013, 2,301 patients in the SEER database were diagnosed with primary EMPD, and 771 of those patients met our inclusion criteria. As shown in Table 1, 510 (66.1%) patients were married, while 180 (23.3%) were divorced, separated, or widowed patients, and only 81 (10.5%) patients had never married. The median age at diagnosis was 68 years old (range 27–100). There were more females (522) than males (249). In total, 76.8% were white or black, and 23.2% were American Indians, AK Natives, or Asian/Pacific Islanders. We divided the primary site into the following six categories: the perianal area, truncal skin, vulva, penis, scrotum, and other. The majority of tumors were located in the vulva (57.7%). In total, 623 (80.8%) patients were diagnosed with localized disease, 132 (17.1%) with regional metastases, and 16 (2.1%) with distant metastases. Surgical treatment was performed in 702 (91.1%) patients, and 49 (6.4%) underwent radiotherapy.

**Table 1:** Clinical characteristics of patients diagnosed with primary EMPD and factors affecting prognosis in primary EMPD patients by univariate and multivariate analyses of data from the SEER database.

**Clinical characteristics**	**No. of patients**	**Univariate analysis**		**Multivariate analysis**		
		**mOS (months)**	***p*-value**	**HR**	**95% CI**	***p*-value**
**Marital status**			<0.001			
Married	510	351.7		1.0		
Other^*^	180	209.0		1.663	1.051–2.633	0.030
Never married	81	307.6		1.969	1.058–3.667	0.033
**Age at diagnosis**			<0.001			
<65	291	381.3		1.0		
65–74	247	253.6		2.453	1.345–4.474	0.003
≥75	233	146.9		5.750	3.224–10.254	<0.001
**Sex**			0.016			
Male	249	243.6		1.0		
Female	522	337.7		0.622	0.287–1.346	0.228
**Race**			0.848			
White/black	592	329.2				
Other^#^	179	324.2				
**Primary site**			<0.001			
Perianal area	25	113.5		1.0		
Truncal skin	152	202.5		0.367	0.166–0.815	0.014
Vulva	445	341.8		0.399	0.158–1.004	0.051
Penis	21	165.9		0.216	0.045–1.023	0.053
Scrotum	111	261.5		0.246	0.107–0.566	0.001
Other	17	118.0		0.617	0.187–2.037	0.428
**SEER historic stage A**			<0.001			
Localized	623	346.0		1.0		
Regional	132	283.3		2.180	1.385–3.431	0.001
Distant	16	46.6		18.821	9.050–39.142	<0.001
**Surgery**			0.001			
No	69	144.1		1.0		
Yes	702	334.9		0.804	0.435–1.485	0.485
**Radiation**			<0.001			
No	722	337.6		1.0		
Yes	49	125.2		2.039	1.124–3.699	0.019

* *Other: divorced, separated, or widowed*.

# *Other: American Indians, AK Natives, or Asian/Pacific Islanders*.

*EMPD, extramammary Paget's disease; HR, hazard ratio; SEER, Surveillance, Epidemiology, and End Results; mOS, median overall survival*.

From 2004 to 2013, 460 patients had TNM stage records based on the sixth edition of the American Joint Committee on Cancer (AJCC) Cancer Staging Manual (Supplemental Tables 1,2). Eighty-two (17.8%) patients were T1 stage, 141 (30.7%) patients were T2 stage, 35 (7.6%) patients were T3 stage, 14 (3.0%) patients were T4 stage, and 188 (40.9%) patients were T stage unknown. Four hundred thirty-five (94.6%) patients were N0 stage, 10 (2.2%) patients were N1 stage, and 15 (3.3%) patients were N stage unknown. Four hundred forty-nine (97.6%) patients were M0 stage, five (1.1%) patients were M1 stage, and six (1.3%) patients were unknown. As for AJCC stage, 75 (16.3%) patients were at stage I, 158 (34.3%) patients were at stage II, 26 (5.7%) patients were at stage III, 11 (2.4%) patients were at stage IV, and 190 (41.3%) patients were unknown.

#### Survival

The disease-specific 5-year survival rates were 87% for all primary EMPD patients, 92% for patients with localized disease, 77% for patients with regional metastases, and 16% for patients with distant metastases.

The factors affecting prognosis determined by univariate and multivariate analyses are presented in Table 1.

**Figure 2:**
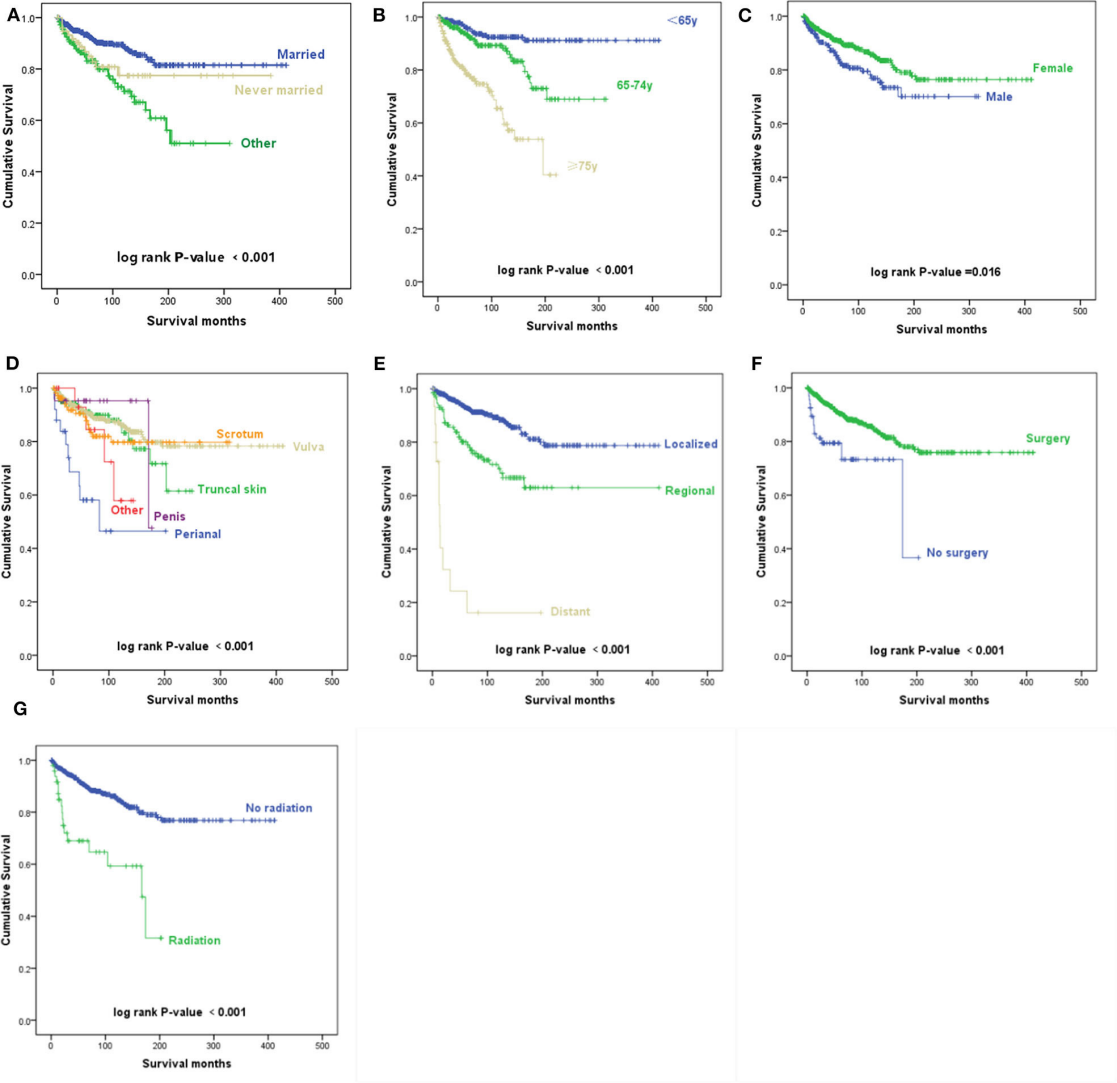
Kaplan–Meier survival curves. **(A)** Kaplan–Meier survival curves according to the marital status. **(B)** Kaplan–Meier survival curves according to the age at diagnosis. **(C)** Kaplan–Meier survival curves according to sex. **(D)** Kaplan–Meier survival curves according to the primary site. **(E)** Kaplan–Meier survival curves according to the Surveillance, Epidemiology, and End Results (SEER) historic stage A. **(F)** Kaplan–Meier survival curves according to surgery. **(G)** Kaplan–Meier survival curves according to radiotherapy.

The Kaplan–Meier survival curves suggest that patients who were divorced, separated, or widowed had significantly worse disease-specific survival than those who were either married or never married (*p* < 0.001) (Figure 2A). We took the factor of age into consideration and performed further analysis toward the influence of marital status. A stratified analysis of marital status counteracting the factor of age suggested that compared with patients with marital status of married, in patients with marital status of divorced, separated, or widowed (HR, 1.409; *p* = 0.159) and never married (HR, 1.612; *p* = 0.107), no statistically significant difference was found.

According to the univariate analyses, older age at diagnosis (*p* < 0.001) (Figure 2B), male sex (*p* = 0.016) (Figure 2C), primary disease in the perianal area (*p* < 0.001) (Figure 2D), distant metastasis (*p* < 0.001) (Figure 2E), and radiotherapy (*p* < 0.001) (Figure 2G) were associated with decreased survival, while patients who received surgery had an improved prognosis (*p* = 0.001) (Figure 2F).

**Figure 3:**
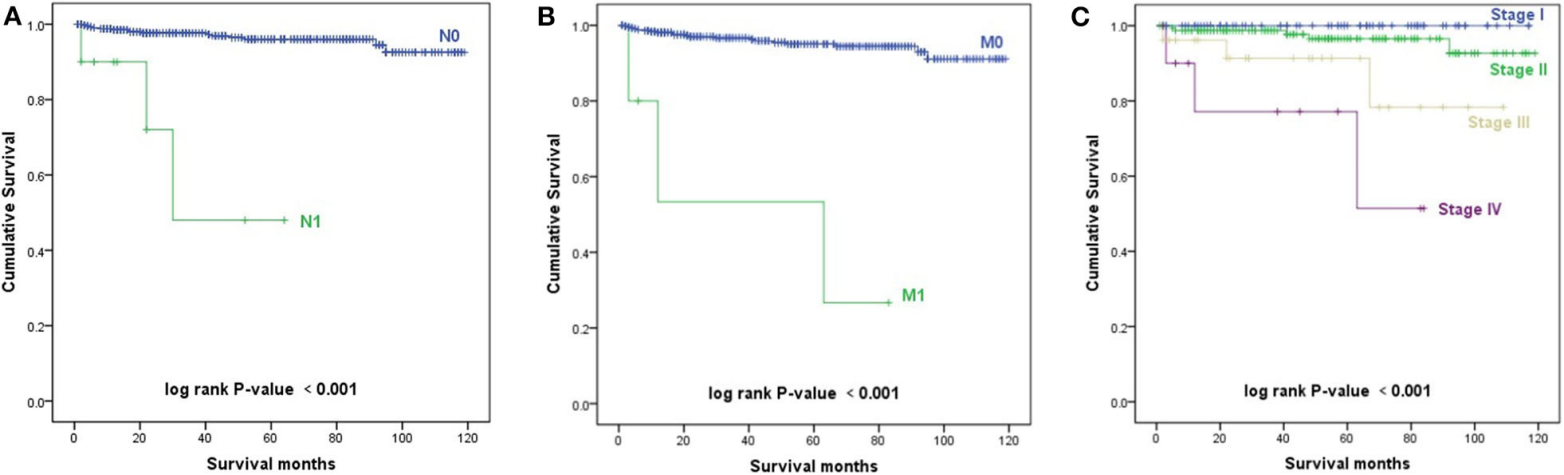
Kaplan–Meier survival curves. **(A)** Kaplan–Meier survival curves according to the N stage. **(B)** Kaplan–Meier survival curves according to the M stage. **(C)** Kaplan–Meier survival curves according to the TNM stage.

The Kaplan–Meier survival curves of AJCC TNM stage are shown in Figure 3. Patients with a higher N stage, M stage, and AJCC stage had a decreased prognosis (*p* < 0.001).

As shown in Table 1, the results of multivariable Cox regression analysis revealed that patients diagnosed at 65–74 years of age (HR, 2.453; *p* = 0.003) and 75 years or older (HR, 5.750; *p* < 0.001) had decreased survival when compared with the survival of patients younger than 65 years. Consistent with the univariate analysis, compared with patients with perianal primary sites, patients with truncal skin primary sites (HR, 0.367; *p* = 0.014) and primary sites in the scrotum (HR, 0.246; *p* = 0.001) had better survival. Patients with distant metastasis (HR, 18.821; *p* < 0.001) and regional metastasis (HR, 2.180; *p* = 0.001) of SEER historic stage A had worse prognoses than patients with localized EMPD. Compared with patients who did not undergo radiotherapy, those who received radiotherapy had decreased survival, with an HR of 2.039 (*p* = 0.019). However, race, sex, and surgery did not influence survival according to our study (*p* > 0.05).

**Figure 4:**
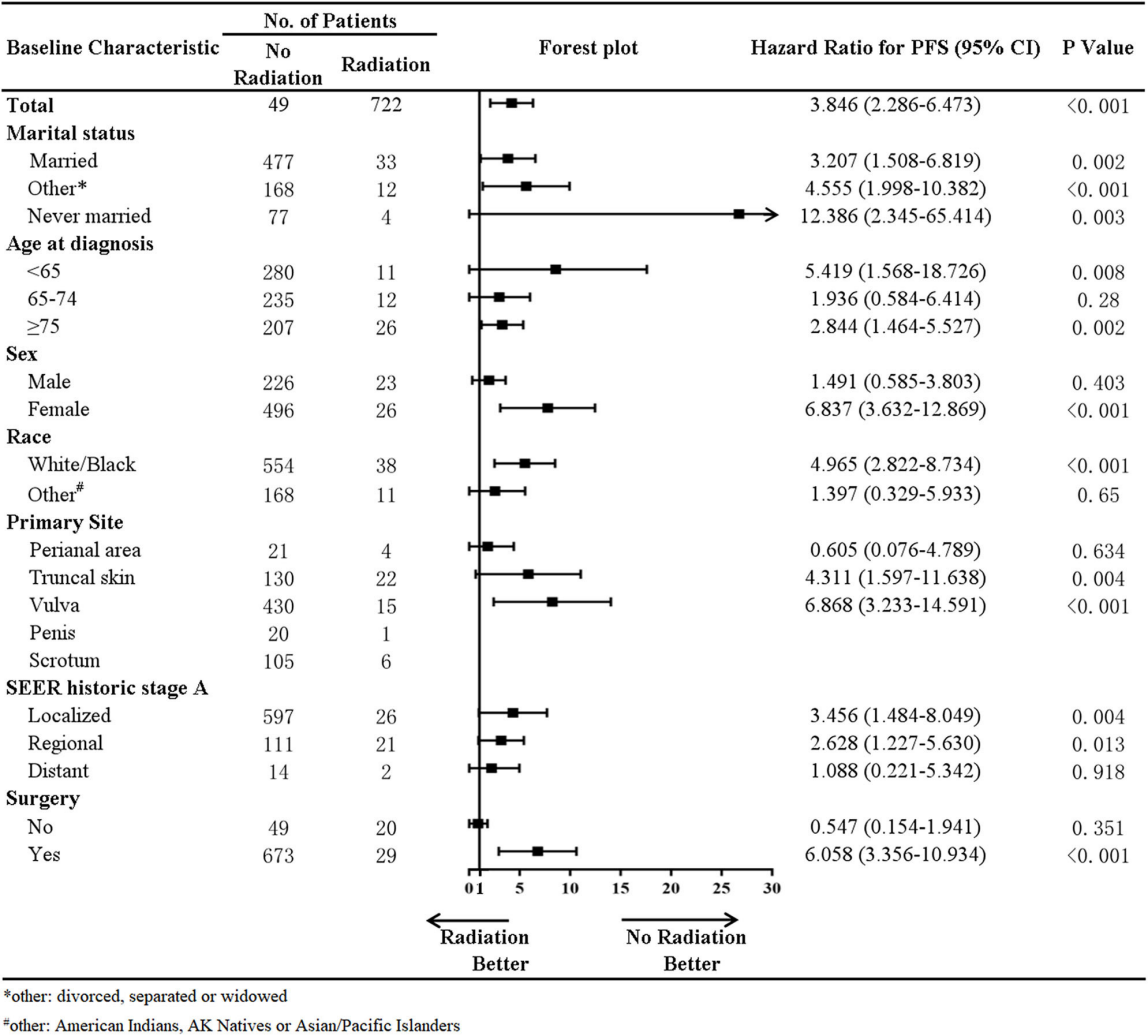
Forest plot of stratified analysis in radiation therapy.

In addition, a stratified analysis of radiation therapy was performed in our study (Figure 4). In patients with a marital status of married (HR, 3.207; *p* = 0.002), divorced, separated, or widowed (HR, 4.555; *p* < 0.001), never married (HR, 12.386; *p* = 0.003), age at diagnosis younger than 65 years old (HR, 5.419; *p* = 0.008), higher than 75 years old (HR, 2.844; *p* = 0.002), primary site at the truncal skin (HR, 4.311; *p* = 0.004) or vulva (HR, 6.868; *p* < 0.001), SEER historic stage A of localized (HR, 3.456; *p* = 0.004) and regional metastases (HR, 2.628; *p* = 0.013), and surgery (HR, 6.058; *p* < 0.001), radiotherapy was associated with decreased survival. In female (HR, 6.837; *p* < 0.001) and White/Black (HR, 4.965; *p* < 0.001) patients, radiotherapy was also associated with decreased survival.

### Data From Primary Extramammary Paget's Disease Patients Recorded in Our Hospital

In total, 44 Chinese primary EMPD patients were analyzed. Their demographic characteristics and clinical characteristics are summarized in Table 2. We found that 10 (22.7%) patients had a history of other neoplasms. It is important to note that pathology confirmed that these tumors were independent of EMPD. All pathological sections were reviewed by at least two experienced pathologists. Erythema (20 cases, 45.4%) was the most common initial presenting symptom.

**Table 2:** The demographic features and clinical characteristics of the patients diagnosed with primary EMPD in our hospital from January 2008 to December 2017.

**Demographic features**	**No. of patients**	**Clinical characteristics**	**No. of patients**
**Sex**		**Presenting symptom**	
Male	37 (84.1%)	Pruritus	9 (20.4%)
Female	7 (15.9%)	Ulcer	5 (11.4%)
**Age at diagnosis (years, mean 69.5, range from 52 to 87)**		Eczema	5 (11.4%)
<60	8 (18.2%)	Erythema	20 (45.4%)
60–69	13 (29.5%)	Phyma	5 (11.4%)
70–79	16 (36.4%)	**Level of invasion**	
≥80	7 (15.9%)	Invasive	16 (36.4%)
**Smoker**		Non-invasive	28 (63.6%)
Non-smoker	28 (63.6%)	**Primary Location**	
Smoker	16 (36.4%)	Penoscrotal	31 (70.5%)
**Drinker**		Vulva	7 (15.9%)
Non-drinker	26 (59.1%)	Others	6 (13.6%)
Drinker	18 (40.9%)	**Size (mean 21.4 cm**^**2**^**, range 0.25–100 cm**^**2**^**)**	
**Tumor history**		≤ 21 cm^2^	18 (40.9%)
None	34 (77.3%)	>21 cm^2^	12 (27.3%)
Yes	10 (22.7%)	NA	14 (31.8%)
Stomach	3 (6.8%)	**Surgery**	
Colon	2 (4.5%)	Curative excision	6 (13.6%)
Lung	2 (4.5%)	Wide local excision	14 (31.8%)
Pancreas	1 (2.3%)	Local excision	22 (50.0%)
Breast	1 (2.3%)	No surgery	2 (4.5%)
Parotid gland	1 (2.3%)	**Recurrence**	
**Delay in diagnosis (mean 3.8 year, range 0.04–35 years)**		Yes	14 (31.8%)
≤ 2 years	26 (59.1%)	No	23 (52.3%)
>2 years	16 (36.4%)	NA	7 (15.9%)
NA	2 (4.5%)		

*EMPD, extramammary Paget's disease*.

**Figure 5:**
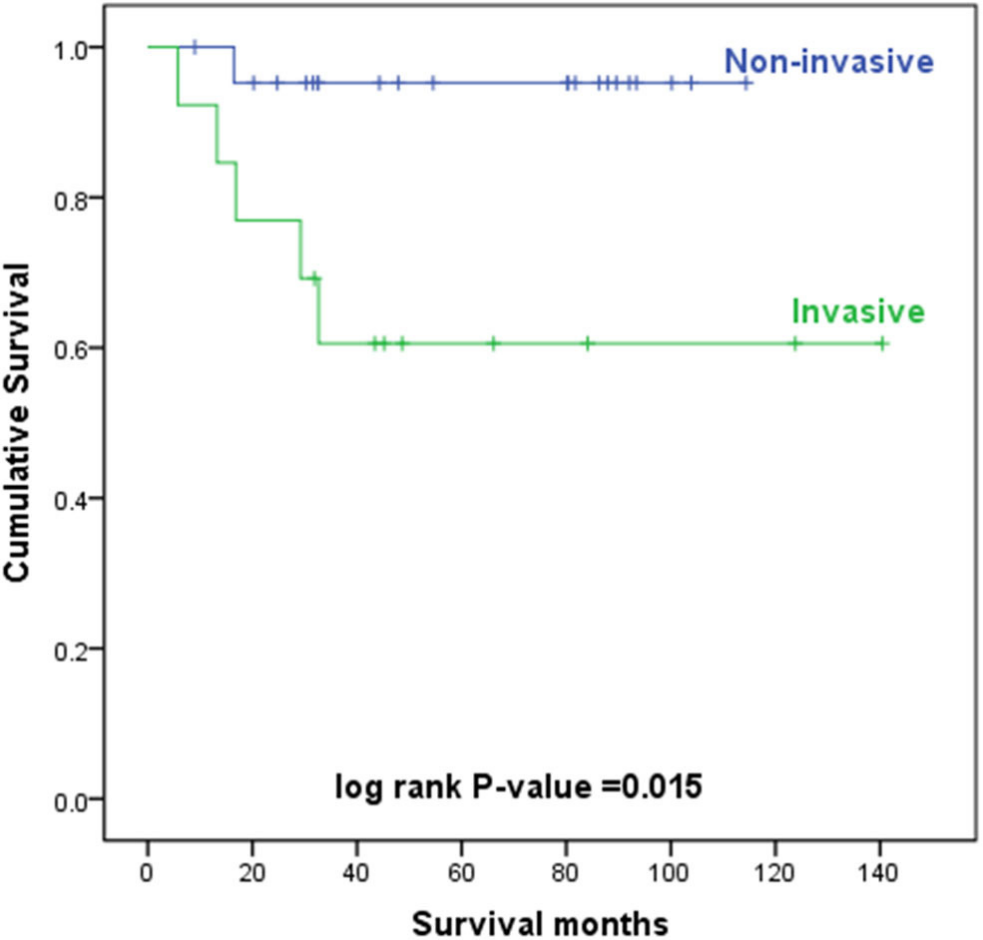
Kaplan–Meier survival curves according to the depth of invasion.

The level of invasion was divided into the following two groups: invasive (Paget cells invaded through the epidermal basement membrane into the dermis) and non-invasive (intraepidermal). Sixteen (36.4%) patients had invasive tumors, and 28 (63.6%) patients had non-invasive tumors. The mean delay in diagnosis (the time from the onset of symptoms until diagnosis) was 3.8 years (ranging from 0.04 to 35). As shown in Table 3, invasive primary EMPD may be related to a positive smoking history (*p* = 0.038) and a longer delay in diagnosis (*p* = 0.005). Patients with invasive primary EMPD (*p* = 0.010) or a delay in diagnosis more than 2 years (*p* = 0.013) might have an elevated rate of disease recurrence. According to the univariate analyses, patients with invasive primary EMPD (Figure 5) were associated with decreased survival (*p* = 0.015). However, factors of sex, age at diagnosis, delay in diagnosis, primary location, and surgical method were not associated with prognosis.

**Table 3:** Differences between invasive and non-invasive primary EMPD and differences between recurrent and non-recurrent primary EMPD in our hospital from January 2008 to December 2017.

**Clinical characteristics**	**EMPD level of invasion**		***p*-value**	**Clinical characteristics**	**EMPD recurrence**		***p*-value**
	**Non-invasive**	**Invasive**			**Non-recurrence**	**Recurrence**	
Case, n	28	16		Case, n	23	14	
**Age at diagnosis**				**Age at diagnosis**			
≤ 65 years	10	6	0.906	≤ 65 years	8	5	1.000
>65 years	18	10		>65 years	15	9	
**Sex**				**Sex**			
Male	23	14	1.000	Male	21	12	0.625
Female	5	2		Female	2	2	
**Smoking history**				**Smoking history**			
Non-smoker	21	7	0.038	Non-smoker	14	9	0.835
Smoker	7	9		Smoker	9	5	
**Alcohol consumption history**				**Alcohol consumption history**			
Non-drinker	18	8	0.354	Non-drinker	11	9	0.330
Drinker	10	8		Drinker	12	5	
**Tumor history**				**Tumor history**			
None	22	12	1.000	None	17	12	0.683
Yes	6	4		Yes	6	2	
**Delay in diagnosis**				**Delay in diagnosis**			
≤ 2 years	21	5	0.005	≤ 2 years	18	5	0.013
>2 years	7	9		>2 years	5	7	
NA	0	2		NA	0	2	
**Size, cm**^**2**^				**Size, cm**^**2**^			
≤ 21 cm^2^	13	5	0.487	≤ 21 cm^2^	12	5	0.681
>21 cm^2^	6	6		>21 cm^2^	6	5	
NA	9	5		NA	5	4	
				**Level of invasion**			
				Invasive	5	9	0.010
				Non-invasive	18	5	

*EMPD, extramammary Paget's disease*.

Most patients (95.5%) underwent surgical excision as the primary treatment. And medical records showed that those patients did not receive other treatments until disease recurrence. Only two patients (4.5%) did not undergo surgery due to old age. One patient received red light therapy, and another received photodynamic therapy (PDT). Fourteen (31.8%) patients experienced recurrence, 23 (52.3%) remained disease-free, and seven were lost to follow-up. Of the 16 patients who had invasive tumors, nine (56.3%) developed local recurrence, and two were lost to follow-up. After recurrence, seven patients underwent reoperation, one patient received radiation therapy, one received PDT, one received chemotherapy, and four patients chose to receive the best supportive care.

Among the 44 primary EMPD patients, 26 (59.1%) had a record of immunohistochemical testing. Among these 26 patients, 23 (88.5%) were cytokeratin (CK)7(+), 14 (53.8%) were CK20(-), 11 (42.3%) were gross cystic disease fluid protein (GCDFP)-15(+), and 18 (69.2%) were CEA(+). In summary, seven (26.9%) patients stained CK7(+) and CK20(-); seven (26.9%) patients stained CK7(+), CK20(-), and GCDFP-15(+); three (11.5%) patients stained CK7(+) and GCDFP-15(+); and three (11.5%) patients stained CK7(+) and GCDFP-15(-). Five patients underwent HER2 testing, and three of the five were strongly positive for HER2(3+). Interestingly, all three of these patients had invasive EMPD.

The 5-year overall survival rates were 81% for all primary EMPD patients, 95% for patients with non-invasive EMPD, and 61% for patients with invasive EMPD in our study.

## Discussion

### Incidence and Clinical Parameters

EMPD is an uncommon malignant tumor and accounts for 6.5% of all cutaneous Paget's disease (7). Previous reports have described that the most common sites are the areas rich in apocrine glands (1–4). The results of the analysis based on patients from our hospital and the SEER program data are consistent with these reports. The mean age at diagnosis was 69.5 years based on data from our center and 67.7 years old based on the SEER program data. Thus, the peak age for the development of primary EMPD appears to be in the 60- to 70-year-old age group. The male-to-female ratio of patients with EMPD is 249:522 in the SEER program and 37:7 based on data from our center. It seems that EMPD is most common in Caucasian women and Asian men, which is in good agreement with literature reports (8–11). Secondary malignancies represent approximately 16% (or one in six) of cancers reported to the National Cancer Institute's SEER Program (12). In the study involving the SEER database, patients with one primary malignant tumor only and EMPD as the first of two or more primary malignant tumors were included. In addition, 25.6% of patients from the SEER program data and 22.7% of patients from our center data had a secondary tumor history. Similarly, it has been reported that patients with primary EMPD are at an increased risk of other malignant neoplasms, mainly anal and colorectal cancers, which are mainly associated with the site of origin of the disease (13). Monitoring to detect the development of other tumors should be conducted in patients with primary EMPD.

### Diagnosis

EMPD is mainly diagnosed by pathological biopsy with hematoxylin–eosin and immunohistochemical staining. There are two forms of EMPD, namely, primary EMPD and secondary EMPD. Primary EMPD originates in the epidermis or apocrine sweat glands and is not associated with an underlying carcinoma, while secondary EMPD is thought to have a relationship with an underlying internal neoplasm (10). Though specific immunohistochemical stains might help distinguish between primary and secondary EMPD, the precise origin of EMPD is still unclear. CK7 is a sensitive marker for EMPD, and CK20 is reported to be present in many carcinomas of the urothelial and gastrointestinal tracts (14). Moreover, GCDFP-15 is strongly expressed in EMPD patients without underlying malignancies (14, 15). In addition, carcinoembryonic antigen (CEA)-negative staining seems to be associated with the presence of underlying carcinomas. Immunohistochemical staining results in our study based on data from our center verified this guideline. Consequently, CK7, CK20, and GCDFP-15 could be used to help distinguish between primary and secondary EMPD. Primary EMPD usually stains CK7(+), CK20(-), and GCDFP-15(+), while secondary EMPD usually stains CK7(+), CK20(+), and GCDFP-15(-).

We found that a significant delay in diagnosis could be observed in EMPD patients according to the present literature and the data from our center. The following reasons could explain this phenomenon. First, the most common initial symptoms, such as pruritus, rash, erythema, erosion, pain, and exudation, are non-specific. It is difficult to distinguish EMPD from Bowen's disease, leukoplakia, squamous cell carcinoma, benign papulosquamous diseases, mycosis fungoides, and melanoma (2). For instance, Lee et al. (16) reported a case of pagetoid Bowen's disease that was initially misdiagnosed as EMPD. In addition, the most common sites affected are the vulva in women and the scrotal, perianal, or penile skin in men (17). Most patients are too shy to seek medical help due to the private nature of the site, and the physical examination of these areas is frequently not as thorough as that of other areas. Furthermore, due to the rarity of EMPD, some young physicians often lack enough awareness of the disease. Thus, a biopsy of skin lesions should be performed in a timely manner if the patient's symptoms are not improved after symptomatic therapy.

### Treatment

The initial treatment of EMPD is radical surgical excision (2, 10, 18, 19). However, the efficacy of radiotherapy is controversial (20–22). In our study, based on data from the SEER program, compared with patients who did not undergo radiotherapy, those who received radiotherapy had a decreased survival rate, with an HR of 2.039 (*p* = 0.019). A stratified analysis of radiation therapy suggested that in patients whose age at diagnosis is younger than 65 years old, higher than 75 years old, primary site at the truncal skin or vulva, and SEER historic stage A of localized and regional metastases, radiotherapy was associated with decreased survival. In female and White/Black patients, radiotherapy was also associated with decreased survival. Thus, radiotherapy was not recommended for the above subgroups. And a study that included 290 patients with EMPD from the National Cancer Database (NCDB) of the United States of America suggested, in line with the SEER analysis, radiotherapy used to be associated with worse survival outcomes in EMPD patients (23). However, Hata et al. (24) reported that radiotherapy was safe and effective and contributed to prolonged survival in patients with primary EMPD. On the one hand, the decreased survival associated with radiotherapy in our study based on data from the SEER program was likely attributable to more advanced and/or recalcitrant disease among these patients. On the other hand, the possible cause of this difference is that the radiation dose, fields, radiation sources, and fractionation schedules vary widely between different studies. The optimal radiotherapy for primary EMPD has not been established (25). In our study, in total, 771 patients from the SEER program, only 49 patients received radiation. It was hard to draw firm conclusions based on the limited data. This problem needs to be validated by large clinical trials.

HER2 overexpression is associated with invasive primary EMPD (4). Three of the five patients who underwent HER2 testing were positive for HER2, and all three HER2-positive patients had invasive primary EMPD based on the data from our hospital. Many studies have indicated that EMPD patients with HER2 overexpression exhibit a good response to trastuzumab combined with chemotherapy or monotherapy (26–29). So we recommend that EMPD patients undergo detection of HER2, and trastuzumab monotherapy or combined chemotherapy could be an option for HER2-positive patients.

### Prognosis

In many solid malignancies, TNM stage is essential for estimating the prognosis of patients. According to the literature, the occurrence of lymph node metastasis and distant metastasis in EMPD patients was 34% to 61% (8, 30, 31). Our study suggested that patients with a higher N stage, M stage, and AJCC stage had a decreased prognosis. However, there was no statistical difference in the effect of T stage on prognosis. The greatest dimension of the tumor was used to classify the primary tumor (T stage) in the current staging system for skin cancer. But some literature suggested that tumor size is not associated with prognosis (8, 30). And in some previous studies, the invasion level of the tumor (8, 30), tumor thickness (32, 33), and lymphovascular invasion (30, 32) were reported to have a correlation with prognosis. Thus, basing on this point, maybe a new T stage classification needs to be established for EMPD. Kuniaki Ohara et al. (34) proposed a disease-specific TNM staging system for EMPD to provide prognostic information to help manage EMPD. They adopted tumor thickness and lymphovascular invasion to classify the primary tumor (T stage), and confirmed that these two factors were associated with worse survival.

Results of our studies suggested that primary site in the perianal area may result in decreased survival. This phenomenon has also been observed in published studies (33, 35). Due to the little subcutaneous fat of this site, the risk of metastasis has been greatly increased even in the patient with early-phase dermal invasion (2, 36, 37).

As mentioned previously, the diagnosis of primary EMPD is often delayed. Our study suggested that a longer delay in diagnosis and positive smoking history might be related to dermal invasion. The exact mechanism underlying the elevated risk of dermal invasion in smokers is unknown. One possible reason is the immunosuppression caused by smoking (38). This finding suggests that doctors should be vigilant when encountering such patients and try to shorten the delay in diagnosis. In addition, such patients need to quit smoking.

Moreover, in our study, dermal invasion might be a critical factor in disease recurrence. Lai et al. (1) also reported 33 EMPD cases and suggested that dermal invasion is associated with local recurrence. This finding implies that patients with invasive primary EMPD need more aggressive therapy and closer follow-up (30).

Following the above discussion, we could summarize the similarities and differences between the patients from the SEER database and our center. The peak age (60–70 years old) for the development of primary EMPD and increased risk of accompanying other malignant neoplasms were similar in both cohorts. As for the male-to-female ratio, EMPD is most common in Caucasian women and Asian men. Regarding prognostic factors, older age at diagnosis and primary site in the perianal area were significantly correlated to decreased survival based on data from the SEER program, while age at diagnosis and primary tumor location were not associated with prognosis based on data from our hospital. However, analysis results of the SEER database were more consistent with the literature reports. This may be related to the limited amount of data from our hospital.

In conclusion, in primary EMPD patients, an older age at diagnosis, primary site in the perianal area, distant metastasis, radiotherapy, and higher N stage, M stage, and AJCC stage may result in decreased survival. In addition, analysis of patients in our center suggested that a longer delay in diagnosis and smoking history may be related to dermal invasion. Dermal invasion may be a critical factor affecting disease recurrence.

## Data Availability Statement

All datasets presented in this study are included in the article/Supplementary Material.

## Author Contributions

SW and NZ contributed conception and design of the study. DL organized the database. DL, NZ, and SW performed the statistical analysis. SW and NZ wrote the first draft of the manuscript. YC, YT, and JC wrote sections of the manuscript. YY conceived and designed the experiments, performed the experiments, authored or reviewed drafts of the paper, and approved the final draft. All authors contributed to manuscript revision and read and approved the submitted version.

## Conflict of Interest

The authors declare that the research was conducted in the absence of any commercial or financial relationships that could be construed as a potential conflict of interest.
