# Supplementary material for: Clinical Characteristics, Treatment, and Prognostic Factors of Patients With Primary Extramammary Paget's Disease (EMPD): A Retrospective Analysis of 44 Patients From a Single Center and an Analysis of Data From the Surveillance, Epidemiology, and End Results (SEER) Database
Source: Front Oncol. 2020 Aug 25;10:1114. doi: 10.3389/fonc.2020.01114 (PMC7477308; doi:10.3389/fonc.2020.01114)
Supplement: Supplementary file 2 [file Table_2.pdf]

Clinical Characteristics, Treatment, and Prognostic Factors of Patients With Primary Extramammary Paget's Disease (EMPD): A Retrospective Analysis of 44 Patients From a Single Center and an Analysis of Data From the Surveillance, Epidemiology, and End Results (SEER) Database

Shanshan Weng[1†], Ning Zhu[1†], Dan Li[1], Yurong Chen[1,2], Yinuo Tan[1], Jiaqi Chen[1] and Ying Yuan[1,3*]

[1] *Department of Medical Oncology, The Second Affiliated Hospital, Zhejiang University School of Medicine, Hangzhou, China,*

[2] *Department of Medical Oncology, Zhuji People's Hospital of Zhejiang Province, Shaoxing, China,*

[3] *Cancer Institute (Key Laboratory of Cancer Prevention and Intervention, Chinese National Ministry of Education; Key Laboratory of Molecular Biology in Medical Sciences, Zhejiang Province, China), The Second Affiliated Hospital, Zhejiang University School of Medicine, Hangzhou, China*

[*]***Correspondence:***

Ying Yuan

yuanying1999@zju.edu.cn

[†]*These authors have contributed equally to this work and share first authorship*

Citation:

Weng S, Zhu N, Li D, Chen Y, Tan Y, Chen J and Yuan Y (2020) Clinical Characteristics, Treatment, and Prognostic Factors of Patients With Primary Extramammary Paget's Disease (EMPD): A Retrospective Analysis of 44 Patients From a Single Center and an Analysis of Data From the Surveillance, Epidemiology, and End Results (SEER) Database. Front. Oncol. 10:1114. doi: 10.3389/fonc.2020.01114

Supplemental Table 2 The Stage Group of the 6th edition AJCC Cancer Staging Manual of EMPD

Stage Group	T stage	N stage	M stage
**0**	Tis	N0	M0
**I**	T1	N0	M0
**II**	T2	N0	M0
	T3	N0	M0
**III**	T4	N0	M0
	Any T	N1	M0
**IV**	Any T	Any N	M1
